# Dentists in the Army and Navy

**Published:** 1916-04

**Authors:** 


### DENTISTS IN THE ARMY AND NAVY.

To the members of the Dental Profession:—At the suggestion of the Legislative Committee, and as directed by the Board of Trustees, we are writing you on what appears to the officers of the National Dental Association, as a very important matter, and we respectfully request that you give it your IMMEDIATE attention.

Our Trustees held a meeting in Cleveland, Ohio, February 8th, at which time, upon invitation of Dr. Hinman, Dr. Brown, Chairman of the Legislative Committee, outlined their plans to secure the legislation previously approved at both the Rochester and San Francisco meetings, as follows:

The Committee thinks it advisable to concentrate its efforts toward raising the status and increasing the efficiency of the Army Dental Corps. This will pave the way for similar legislation for the Navy Dental Corps, and at the same time, accord to our profession a more dignified recognition. The efforts thus far have been largely directed in securing the support of those in authority, such as the Secretary of War, the Chief of Staff, the Surgeon General, and the Chairman of the Senate and House Military Affairs Committees, to incorporate our recommendations in their general defense program. Our Committee is confident that they have made positive progress in this direction, but at the same time they feel the NECESSITY FOR BRINGING AT ONCE EVERY POSSIBLE INFLUENCE upon the members of both the Senate and House Military Affairs Committees, in order to make certain that our recommendations will be incorporated in their general defense bill, and not be eliminated by any Conference Committee. Press reports indicate that these bills are to be submitted to Congress before March first.

Upon request of the Chairmen of the Senate and House Military Affairs Committees, Doctors Brown and Gifford, representing the Legislative Committee of our Association, appeared before the Senate Committee February 2nd and the House Committee February 3rd, and Dr. Tignor, of the Army Dental Corps, appeared with them before the House Committee.

In order to acquaint you with the facts in the briefest possible manner regarding the suggestions of our Committee, we are herewith enclosing the specific recommendations submitted to the officials above designated and incorporated in the hearings before both Military Committees. From this you can readily secure data from which you can make an appeal to your Senators and Representatives and urge them to promptly see such members of the Senate and House Military Affairs Committees as they can reach, especially the Chairmen, and request that all use their influence to the end that the recommendations of our Association are incorporated in the general defense bill, and further request that they give this matter their support in every possible way. This is meritorious legislation, as has been adequately demonstrated by the present European war. You might call attention to the fact that members of Congress can secure further arguments by referring to the hearings before the Senate and House Military Affairs Committees as above noted.

We trust that your Society, through yourself and the other officers, will promptly comply with the requests made in this letter. We also suggest that you furnish the Chairman, Dr. Homer C. Brown, 185 E. State St., Columbus, Ohio, with copies of all such letters as may, in your judgment, be of assistance to the Legislative Committee.

In behalf of the National Dental Association, I thank you in advance for your co-operation in this matter.

OTTDL KING, *Gen. Secy.*

P. S.—Telegraph your Senators to request Senator Chamberlain to incorporate in his defense bill, the recommendations of the Legislative Committee of the National Dental Association. THE RESPONSIBILITY IS YOURS. MAY WE DEPEND ON YOUR IMMEDIATE ACTION?

The Army and Navy Register of January 29th, page 132, first column, detailing the hearing of General Gorgas, Surgeon General of the Army, before the House of Representatives Military Committee, has the following to say:—

* * * * "Information was sought concerning the Dental Corps, and it was announced (by General Gorgas) that the new legislation, approved by the War Department, contemplated promotion for Dental Surgeons up to and including the grade of Major; that there was increasingly important functions for Dentists in the military service; that the Dentists were doing splendid work in Europe; that there was an important function in Dental surgery on account of special wounds. General Gorgas added that on a recent trip to Canada he had occasion to inspect a new command destined for service in Europe and found it equipped with an excellent Dental Department and that men were now being accepted for service, despite Dental defects which hitherto would have resulted in their rejection.''

The following recommendations have been made to the Secretary of War by the Legislative Committee of the National Dental Association:

Complying with your request in our interview this morning, we, the Legislative Committee of the National Dental Association, submit and recommend the following in order to increase the efficiency of the Dental Corps of the Army and secure a more equitable recognition of the Dental profession in matters of health and the military status of the Dental Corps:

1. A Dental Reserve Corps, similar to the Medical Reserve Corps, to replace the present ''Acting Dental Surgeons,'' except that members of the Dental Reserve Corps shall be obligated to serve when called upon for active duty.

2. The Dental Corps to consist of:

(a) First Lieutenants, to be appointed from the Dental Reserve Corps after two years active service, who shall be not less than 23 nor more than 30 years of age, and otherwise qualified as at present.

(b) Captains, who shall be promoted to this grade after seven years of total service, including service in Dental Reserve Corps and as Contract Dental Surgeon, Dental Surgeon, or Acting Dental Surgeon.

(c) Majors, not to exceed 25 per cent. of the Dental Corps, to be promoted according to seniority, as in the Medical Corps.

(d) A Colonel, who shall be Chief of the Dental Corps, acting under the direction of the Surgeon General, to be appointed by the President, by and with the consent of the Senate, for a period of four years. The first Colonel to be appointed from the fifteen senior officers of the Dental Corps, after which they shall be appointed from the Majors of this Corps.

3. The strength of the Dental Corps to be as now provided (1 to 1,000 of the enlisted strength).

4. The service heretofore rendered as Dental Surgeon, Contract Dental Surgeon, or Acting Dental Surgeon, shall be computed as commissioned service.

5. The right to command shall be limited to the members of the Dental Corps and the Dental Reserve Corps and to enlisted men serving as their assistants.

Respectfully submitted,

Legislative Committee of the National Dental Association.

